# Advancing Knowledge on the Environment and Its Impact on Health, and Meeting the Challenges of Global Environmental Change

**DOI:** 10.1289/ehp.1206118

**Published:** 2012-12-03

**Authors:** Fan Wu

**Affiliations:** Shanghai Municipal Center for Disease Control and Prevention Shanghai, China, E-mail: fwu@scdc.sh.cn

During the past century, accelerated urbanization and industrialization across the world have created environ-mental problems that not only threaten our future but also affect our health now.

The World Health Organization (WHO 2006) estimated that 24% of the disease burden (healthy life-years lost) and 23% of all deaths (pre-mature mortality) were attributable to environ-mental factors. Among children < 14 years of age, 36% of deaths were attributed to environmental factors. Compared with people in developed countries, people in developing countries are exposed to higher levels of pollution and have reduced access to health care. This may explain why 25% of deaths in developing countries (vs. 17% in developed countries) can be attributed to environmental factors. The number of years of healthy life lost due to environmental risk factors is about 15 times higher in developing countries (and up to 120–150 times in some locations) than in developed countries. The WHO (2009) attributed approximately 2.4 million deaths in China in 2004 to environmental causes. For every 1,000 Chinese, there was an average reduction of 32 years of healthy living, and the environmental burden of disease was responsible for 21% of the entire disease burden.

China is the largest developing country in the world because its industrialization and urbanization started later than in most developed nations. At present, development in China is occurring at a rapid pace, which is placing great pressure on environ-mental protection and resource management in the nation. With globalization and free trade, rapid economic growth is also straining natural resources and increasing global energy consumption. In recent years, the effect China’s develop-ment has on the global environment has become an inter-national concern.

The Chinese government has recently set forth goals to promote the harmonious development of humans and nature, and to improve the quality of the environment, taking into account economic growth. It plans to restructure and prioritize its industries to build a resources-efficient and environmentally friendly society by 2020. The Chinese government also plans to establish a framework to coordinate environmental protection and economic growth. The Chinese government also realizes that China has its own unique set of circumstances and that the best way to protect the environment is to constantly explore and experi-ment with new approaches.

Over the past 50 years, and particularly over the past decade, health conditions for Chinese have improved significantly. However, disease types and patterns have shifted. Chronic and non-communicable diseases have become the predomi-nant disease burdens and are the leading causes of death in China. Environmental factors are one of the main reasons for changes in disease patterns. As well as modern metropolises, such as Beijing, Shanghai, and Guangzhou, China has less developed areas in the central and western parts of the country. Thus, China is somewhat unique in that it faces environmental health issues for both developed and developing living conditions.

During the rapid global growth of industrialization and urbani-za-tion, there have been many reports of environmental incidents and pollution. From elevated blood lead levels in children and high levels of melamine in baby formula powder in China, to the earthquake and tsunami in Japan and subsequent human exposures to chemicals and radio-activity, environment-related incidents have become significant concerns for governments and the public.

Because of the seriousness and frequency of incidents that affect the environment, the public has become more and more concerned about effects on long-term health, especially the health of children. Governments need to devise strategies to pro-actively protect the environment. In addition, governments should promote public awareness of the environment and its impact on human health. This includes encouraging the public to be better informed about the environment, raising public awareness of environmental protection, and convincing people to use their knowledge of environment and healthy living practices to avoid environmental risks. In doing so, we can protect the environment and also improve public health while fostering a healthy and sustainable environ-ment that is beneficial for human develop-ment.

Disseminating accurate and reliable information about the effects of the environment on human health is a goal of scientific journals such as *Environmental Health Perspectives* (*EHP*). In 2001, *EHP* published its first Chinese Edition to reach a growing Asian population interested in human environmental health sciences. Since 2004, the Shanghai Municipal Center for Disease Control and Prevention (SCDC) and *EHP* have been collaborating to publish the *Chinese Edition, Environmental Health Perspectives* (*CEHP*), which has opened a window for Chinese people to learn about cutting-edge research and current, accurate news in the field of environmental human health. Currently, *CEHP* has an extensive readership in China. Its readers include not only government officials at various levels from different departments but also professionals in the fields of preventive medicine, life sciences, environmental protection, environmental medicine, and environmental policy. *CEHP* has also become a reference source for research and teaching professionals in the field of public health and for anyone promoting environmental conservation and its positive impact on human health.

In the years to come, we aim to fully use the SCDC–*EHP* collaboration to keep professionals and the public abreast of the most current international trends in environmental health, as well as cutting-edge research methods and technologies, theories, practices, and policies. We envision using the SCDC–*EHP* platform to narrow the gap for work in the environmental health field between China and its inter-national counter-parts, to help protect the health of Chinese citizens, and to facilitate economic and social development in China.
